# Association Between Serum Cortisol Levels and Clinical Outcomes in Acute Ischemic Stroke: A Prospective Observational Study

**DOI:** 10.7759/cureus.95325

**Published:** 2025-10-24

**Authors:** M Ishaivanan, M C Vinatha, V Padma, S V Sathyapriya, Heshish Reddy, Lakshmi Chaitanya Varma Pusapati, Sharath Nallaperumal

**Affiliations:** 1 Department of General Medicine, Sree Balaji Medical College and Hospital, Chennai, IND

**Keywords:** acute ischemic stroke, prognosis, serum biomarker, serum cortisol, stroke severity

## Abstract

Background

Acute ischemic stroke (AIS) triggers activation of the hypothalamic-pituitary-adrenal (HPA) axis, resulting in elevated cortisol levels. Cortisol influences glucose mobilization, cardiovascular output, and metabolic responses. This study aimed to evaluate the relationship between serum cortisol levels and outcomes in patients with AIS.

Methods

This prospective analytical study was conducted in the Department of Internal Medicine, Sree Balaji Medical College and Hospital, Chennai, over 12 months (April 2023-March 2024). A total of 75 patients presenting with AIS within 72 hours of onset were included. Stroke severity was assessed using the National Institutes of Health Stroke Scale (NIHSS), and serum cortisol levels were measured on admission. Functional outcome was evaluated using the Modified Rankin Scale (mRS) at 15 days. Statistical analysis included Student’s t-test, ANOVA, Mann-Whitney test, and Pearson correlation.

Results

The mean age of participants was 59.2 ± 16.4 years, with a male predominance (57.3%). Common comorbidities included hypertension (37.3%), diabetes mellitus (26.7%), and coronary artery disease (34.7%). The average NIHSS score was 10.61, indicating moderate stroke severity. Elevated cortisol levels were strongly correlated with higher NIHSS and worse mRS scores. Patients with cortisol levels >690 nmol/L had more severe strokes, poorer functional outcomes, and higher mortality. Non-survivors demonstrated significantly higher mean cortisol levels (985.8 nmol/L) compared to survivors (726.5 nmol/L). Cortisol elevation also showed associations with hyperglycemia and hypercholesterolemia.

Conclusion

Serum cortisol is a reliable prognostic marker in AIS, closely linked to stroke severity, functional outcome, and mortality. Incorporating cortisol measurement into routine evaluation may improve prognostic accuracy and help guide management strategies for better patient care.

## Introduction

Acute ischemic stroke (AIS) is a neurological emergency caused by a sudden interruption of cerebral blood flow, resulting in neuronal injury and functional impairment [1]. It accounts for nearly 87% of all strokes and remains a leading cause of death and long-term disability worldwide [1-3]. According to the World Health Organization, stroke is responsible for approximately 11% of global deaths [2]. In India, recent estimates from the Global Burden of Disease (GBD) Study and the Indian Council of Medical Research report an age-adjusted incidence of 119-145 per 100,000 population, with stroke contributing to nearly 7% of all deaths nationally [4,5]. Beyond its health burden, AIS imposes a substantial socioeconomic impact through prolonged hospitalization, rehabilitation needs, and loss of productivity. Early identification of patients at risk for severe outcomes and accurate prognostication are therefore essential for guiding timely interventions and optimizing resource utilization.

Traditional vascular risk factors, such as hypertension, diabetes mellitus, atrial fibrillation, and dyslipidemia, are well-established predictors of stroke occurrence and outcomes. However, these factors alone do not account for the full variability observed in stroke prognosis [5]. This has prompted growing interest in identifying reliable biochemical markers that could complement clinical and imaging assessments. Among these, serum cortisol has emerged as a promising candidate due to its central role in the stress response and its potential prognostic significance in AIS [4,5].

Cortisol, the primary glucocorticoid secreted by the adrenal cortex under regulation of the hypothalamic-pituitary-adrenal (HPA) axis, modulates metabolism, immune function, and cardiovascular homeostasis [3,4]. In the context of AIS, activation of the HPA axis results in elevated cortisol levels that help sustain energy demands during acute stress but may also exacerbate deleterious processes such as hyperglycemia, hypertension, immune suppression, and impaired recovery [4]. Recent studies and meta-analyses have demonstrated that elevated admission cortisol levels are associated with larger infarct size, greater neurological deficit as measured by the National Institutes of Health Stroke Scale (NIHSS) [6], poorer functional outcomes based on the Modified Rankin Scale (mRS) [7], and higher short-term mortality [8,9]. However, whether cortisol actively contributes to poorer outcomes or merely reflects the systemic stress response remains uncertain.

Although multiple international studies support the association between cortisol and stroke outcomes, results have not been entirely consistent, and prospective data from South Asian populations remain limited. Only a few Indian studies have explored this relationship, underscoring the need for region-specific data to improve prognostic applicability in local settings [10,11].

Given this background, the present study was designed to evaluate the relationship between serum cortisol levels and both stroke severity and outcome in AIS. The primary objective of this study was to assess the relationship between serum cortisol levels and stroke severity, as measured by the NIHSS, and functional outcomes, as assessed by the mRS, at 15 days post-admission. The secondary objective was to evaluate the association between serum cortisol levels and in-hospital mortality among patients with AIS. The study was based on the hypothesis that higher admission serum cortisol levels are associated with greater stroke severity and poorer short-term functional outcomes.

## Materials and methods

This prospective analytical study was conducted in the Department of Internal Medicine, Sree Balaji Medical College and Hospital, Chennai, from April 2023 to March 2024. Patients were recruited from the medical wards and the stroke unit. Individuals aged ≥ 18 years presenting with AIS confirmed by brain CT scans and admitted within 72 hours of symptom onset were eligible for inclusion.

Written informed consent was obtained directly from each patient or, in cases of impaired consciousness, from their legally authorized representative or close relative prior to enrollment. Patients were excluded if they were pregnant, had hepatic disease, malignancy, acute febrile illness, hemorrhagic stroke, recent major surgery (within three weeks), or were receiving immunosuppressants, corticosteroids, rifampicin, or phenytoin. 

The sample size was calculated based on prior evidence indicating that approximately 66% of AIS patients with poor outcomes exhibit hypercortisolemia [5]. Assuming a prevalence < 50% among patients with good outcomes, 74 subjects were required to achieve 80% power with a two-sided α = 0.05. A consecutive sampling method was employed until the target was reached, resulting in 75 patients included in the final analysis.

Detailed demographic data, comorbidities, clinical presentation, and medication history were collected. Stroke severity was assessed on admission using the NIHSS, a validated and publicly available tool [6]. All scoring was performed by trained physicians certified in stroke assessment, and inter-observer consistency was periodically verified by senior neurologists.

Fasting venous samples for serum cortisol estimation were collected between 8:00 a.m. and 9:00 a.m. following an overnight fast of at least eight hours to minimize diurnal variation. Serum cortisol concentrations were measured using a chemiluminescent immunoassay (CLIA) on an Abbott Architect i1000SR analyzer, with a morning reference range of 5-25 µg/dL (Abbott Laboratories, Abbott Park, IL).

All participants underwent CT brain imaging to confirm the diagnosis. Functional outcomes were evaluated on day 15 using the mRS [7]. Blood pressure was measured on admission and classified according to the Joint National Committee (JNC)-7 guidelines [8] as normal (<120/<80 mmHg), pre-hypertension (120-139/80-89 mmHg), Stage 1 hypertension (140-159/90-99 mmHg), and Stage 2 hypertension (≥160/≥100 mmHg). Participants with incomplete or missing cortisol, NIHSS, or mRS data were excluded from the final analysis; no imputation was performed.

Data were anonymized and entered into a secure database with routine validation to ensure accuracy. Descriptive statistics were reported as mean ± standard deviation, median (interquartile range), or frequency (%). For normally distributed variables, comparisons were performed using the Student’s t-test (two groups) and ANOVA (three or more groups); non-normal data were compared using the Mann-Whitney U test. Pearson’s correlation coefficient (r) assessed associations between cortisol levels, NIHSS, mRS, and biochemical parameters. Multivariable linear regression was used to adjust for potential confounders, including age, sex, comorbidities, fasting glucose, and infarct size. All analyses were conducted using SPSS version 25.0 (IBM Corp., Armonk, NY, USA).

Ethical approval was obtained from the Institutional Ethics Committee of Sree Balaji Medical College and Hospital (002/SBMCH/IHEC/2022/1839). The study followed the Indian Council of Medical Research guidelines and the Declaration of Helsinki.

## Results

A total of 75 patients with AIS were included in the study. The demographic characteristics, clinical features, laboratory parameters, imaging findings, stroke severity, functional outcomes, and their association with serum cortisol levels are presented below. Data are expressed as n (%) for categorical variables and mean ± standard deviation for continuous variables.

Demographic and clinical characteristics

The mean age of the study population was 59.2 ± 16.4 years (range: 30-88 years). A total of 41 (54.7%) patients were elderly (≥60 years), while 13 (17.3%) belonged to the younger age group of 30-40 years. The majority of patients were male (n=43; 57.3%). With respect to comorbidities, 20 (26.7%) were known cases of type 2 diabetes mellitus, 28 (37.3%) were hypertensive, and 26 (34.7%) had a history of coronary artery disease (Table 1).

**Table 1:** Baseline demographic and clinical characteristics of the study population (n = 75) CAD: Coronary artery disease

Variable	Category	n (%)
Age (years)	30–40	13 (17.3)
	41–50	8 (10.7)
	51–60	13 (17.3)
	61–70	21 (28.0)
	>70	20 (26.7)
Gender	Male	43 (57.3)
	Female	32 (42.7)
Diabetes mellitus	Present	20 (26.7)
	Absent	55 (73.3)
Hypertension	Present	28 (37.3)
	Absent	47 (62.7)
CAD	Present	26 (34.7)
	Absent	49 (65.3)

Blood pressure profile

The mean systolic blood pressure (SBP) was 141.5 ± 22.3 mmHg (range 90-202 mmHg), and the mean diastolic blood pressure (DBP) was 88 ± 10.2 mmHg (range 64-108 mmHg), providing an overall estimate of the cohort’s hemodynamic status at presentation. A total of 41 (54.7%) patients presented with SBP > 140 mmHg, and 33 (44.0%) had DBP > 90 mmHg. Based on the JNC-7 classification, six (8.0%) patients were normotensive, 15 (20.0%) had pre-hypertension, 37 (49.3%) had Stage 1 hypertension, and 17 (22.7%) had Stage 2 hypertension (Table 2).

**Table 2:** Blood pressure profile of the study population (n = 75) SBP: systolic blood pressure; DBP: diastolic blood pressure; HTN: hypertension, JNC-7: Joint National Committee-7 [8]

Variable	Category	n (%)
SBP (mmHg)	<120	15 (20.0)
	120–140	19 (25.3)
	141–160	27 (36.0)
	161–180	11 (14.7)
	>180	3 (4.0)
DBP (mmHg)	<80	16 (21.3)
	81–90	26 (34.7)
	91–100	20 (26.7)
	>100	13 (17.3)
JNC-7 Classification [8]	Normal (<120/<80)	6 (8.0)
	Pre-hypertension (120–139/80–89)	15 (20.0)
	Stage 1 HTN (140–159/90–99)	37 (49.3)
	Stage 2 HTN (≥160/≥100)	17 (22.7)

Stroke characteristics

CT findings revealed that most patients (n= 65; 86.7%) had middle cerebral artery (MCA) territory infarcts, while seven (9.3%) had anterior cerebral artery (ACA) and three (4.0%) had posterior cerebral artery (PCA) infarcts. The mean NIHSS score at admission was 10.6 ± 8.6 (median: 9, range: 1-41). A total of 38 (50.7%) patients presented with moderate stroke (NIHSS 5-15), 20 (26.7%) with minor stroke, 11 (14.7%) with moderate to severe stroke, and six (8.0%) with severe stroke. Functional status assessed by the mRS showed a mean score of 3.4 ± 1.37. At 15-day follow-up, 26 (34.7%) had a good outcome (mRS 1-2), 42 (56.0%) had a poor outcome (mRS 3-5), and seven (9.3%) patients died (Table 3).

**Table 3:** Stroke characteristics and functional outcomes (n = 75) ACA: anterior cerebral artery; MCA: middle cerebral artery; PCA: posterior cerebral artery; NIHSS: National Institutes of Health Stroke Scale [6]; mRS: modified Rankin Scale [7]

Variable	Category	n (%)
Infarct territory	ACA	7 (9.3)
	MCA	65 (86.7)
	PCA	3 (4.0)
NIHSS score [6]	1–4 (minor)	20 (26.7)
	5–15 (moderate)	38 (50.7)
	16–20 (moderate-severe)	11 (14.7)
	21–42 (severe)	6 (8.0)
mRS score [7]	1	1 (1.3)
	2	25 (33.3)
	3	16 (21.3)
	4	15 (20.0)
	5	11 (14.7)
	6 (death)	7 (9.3)

Biochemical profile

The mean random blood sugar (RBS) was 162.5 ± 71.8 mg/dl, with 27 (36.0%) patients showing hyperglycemia (RBS >200 mg/dl). The mean total cholesterol was 220.3 ± 51.1 mg/dl, with 41 (54.7%) patients showing hypercholesterolemia (>200 mg/dl) (Table 4).

**Table 4:** Biochemical characteristics of the study population (n = 75) RBS: random blood sugar

Variable	Category	n (%)
RBS (mg/dl)	<200	48 (64.0)
	≥200	27 (36.0)
Total cholesterol (mg/dl)	<200	34 (45.3)
	≥200	41 (54.7)

Association between serum cortisol and clinical variables

Serum cortisol levels showed a strong positive correlation with stroke severity and outcome. Cortisol was significantly correlated with NIHSS (r = 0.628, p <0.001), mRS (r = 0.707, p <0.001), random blood sugar (RBS) (r = 0.800, p <0.001), and total cholesterol (r = 0.775, p <0.001) (Table 5). Patients with higher NIHSS scores had significantly elevated cortisol levels, with mean values rising progressively from 462.2 nmol/L in minor stroke (n=20; 26.7%) to 1031.5 nmol/L in moderate-to-severe stroke (n=11; 14.7%) (p <0.001, ANOVA). Similarly, patients with poor outcomes (n=42; 56.0%) had significantly higher cortisol levels (864.9 nmol/L) compared to those with good outcomes (n=26; 34.7%) (p <0.001).

**Table 5:** Correlation and association of serum cortisol levels with clinical variables (n = 75) Statistical test: Pearson’s correlation coefficient (r), with corresponding t-values and p-values. SBP: systolic blood pressure; DBP: diastolic blood pressure; NIHSS: National Institutes of Health Stroke Scale [6]; mRS: modified Rankin Scale [7]; RBS: random blood sugar. *Statistically significant.

Variable	Correlation With Cortisol (r)	Test Statistic (t)	p-value
Age	0.050	0.43	0.671
SBP	0.017	0.15	0.888
DBP	-0.097	-0.83	0.410
NIHSS [6]	0.628	6.89	<0.001*
mRS [7]	0.707	8.54	<0.001*
RBS	0.800	11.39	<0.001*
Total cholesterol	0.775	10.48	<0.001*

Patients with hypercortisolemia (>690 nmol/L; n=49; 65.3%) had significantly higher NIHSS and mRS scores compared with those with normal cortisol levels (≤690 nmol/L; n=26; 34.7%) (p <0.001 for both). Mortality analysis showed that non-survivors (n=7; 9.3%) had significantly higher mean cortisol levels (985.8 nmol/L) compared to survivors (n=68; 90.7%) (726.5 nmol/L, p = 0.019).

Overall, the study demonstrated that elevated serum cortisol levels were consistently associated with higher stroke severity, poor functional outcomes, hyperglycemia, hypercholesterolemia, and increased mortality. The findings highlight the potential utility of serum cortisol as a prognostic biomarker in AIS.

## Discussion

This study demonstrated that elevated serum cortisol levels were consistently associated with greater stroke severity, poorer functional outcomes, and higher mortality among patients with AIS. These findings align with prior reports suggesting that hypercortisolemia reflects both the physiological stress response and the extent of cerebral injury rather than being a direct causal factor. The results reinforce the hypothesis that activation of the HPA axis influences early stroke outcomes. Patients with hypercortisolemia exhibited significantly higher NIHSS scores on admission and poorer functional outcomes at follow-up, as measured by the mRS. Mortality was also higher among patients with markedly elevated cortisol levels, underscoring the prognostic value of this biomarker in stroke care [9,10].

The mean age of participants in this study was 59.2 years, which is consistent with the findings of Ilanchetchenni et al. [11] (58.5 years) and Iranmanesh et al. [12] (64.7 years), though lower than that reported by Agarwal et al. (72.8 years) [13]. Such demographic variation highlights regional differences in stroke epidemiology and risk factor profiles. The relatively younger age distribution in our cohort may be attributed to an earlier onset of vascular risk factors such as diabetes, hypertension, and coronary artery disease, which were common comorbidities among the patients studied.

Cortisol dynamics in this study further reinforced its prognostic value. Patients with poor outcomes had mean cortisol levels of 864.9 nmol/L, while non-survivors exhibited levels as high as 985.8 nmol/L. These values exceeded those reported by Ilanchetchenni et al. [8] (637.1 nmol/L) but followed the same upward trend described by Agarwal et al. [13] and Jnanendrappa et al. [14], both of whom reported a positive correlation between cortisol levels and stroke severity [5,8,11]. In our cohort, elevated cortisol was strongly correlated with higher NIHSS and mRS scores (r = 0.628 and 0.707, respectively), reinforcing its role as a marker of neurological injury and poor recovery potential.

The prognostic role of cortisol has been consistently highlighted in prior literature. Christensen et al. [15] and Marklund et al. [16] found that serum cortisol predicted both stroke severity and long-term mortality, while Fatima et al. [17] demonstrated its association with functional decline. Our findings align with these results, as non-survivors in the present study had markedly higher cortisol levels and NIHSS scores compared with survivors (p = 0.019). Taken together, these findings and those summarized in Table 6 provide strong evidence that hypercortisolemia reflects a maladaptive stress response through HPA axis activation, thereby worsening cerebral injury and systemic metabolic stress. However, not all studies have demonstrated a consistent relationship; Saini et al. [18] questioned whether cortisol independently predicts outcomes once stroke severity is considered, suggesting it may serve more as a surrogate marker of neurological insult than as a causative factor. Such discrepancies highlight the need for larger, multicenter studies to clarify whether cortisol is directly involved in worsening stroke outcomes or primarily reflects the severity of the underlying insult.

**Table 6:** Representative studies on serum cortisol and outcomes in acute ischemic stroke NIHSS: National Institutes of Health Stroke Scale; mRS: modified Rankin Scale This table summarizes key representative studies illustrating the relationship between serum cortisol and stroke outcomes. It is not an exhaustive list of all available literature.

Author (Year)	Sample Size (n)	Mean Age (years)	Key Findings on Cortisol	Outcome Association
Agarwal et al. (2018) [13]	50	72.8 ± 12.5	Higher cortisol linked to severe stroke	Increased mortality, larger lesion size
Ilanchetchenni et al. (2024) [11]	70	58.5 (40–88)	Mean cortisol 637 nmol/L	Correlated with stroke severity and mortality
Iranmanesh et al. (2017) [12]	60	64.7 ± 8.7	Cortisol correlated with NIHSS	Poor functional outcome
Jnanendrappa et al. (2021) [14]	80	61.2 ± 10.3	Elevated cortisol in severe strokes	Poor outcome correlation
Christensen et al. (2021) [15]	95	66.4 ± 11.9	Cortisol predicted stroke severity	Increased mortality
Marklund et al. (2022) [16]	120	68.5 ± 9.8	Higher cortisol in severe cases	Functional decline, higher death rates
Fatima et al. (2023) [17]	100	62.1 ± 12.4	Cortisol correlated with NIHSS and mRS	Worse functional outcomes

Overall, the present study supports the growing consensus that serum cortisol is an important biomarker for risk stratification in AIS. Its strong correlation with NIHSS, mRS, and mortality suggests potential utility in early identification of high-risk patients who may benefit from closer monitoring and more aggressive therapeutic strategies. Incorporating cortisol testing into standard clinical evaluation could improve prognostic assessments and enable individualized treatment strategies aimed at improving functional outcomes and survival [19].

This study has several important limitations. The relatively small sample size (75 patients) and single-center design limit the generalizability of findings to broader populations. Cortisol was measured only once, between 8:00 and 9:00 a.m., and therefore does not account for diurnal or stress-related fluctuations that could influence levels. The 15-day follow-up captures only short-term outcomes and may underestimate delayed recovery or mortality. Additionally, we did not measure other stress-related biomarkers such as ACTH, catecholamines, or inflammatory markers, which could provide mechanistic insight. Potential confounding variables - including comorbidities, concurrent medications, and infarct characteristics - may have influenced cortisol levels and outcomes despite multivariable adjustment. These factors should be considered when interpreting our results, which are exploratory and hypothesis-generating rather than confirmatory.

Despite these limitations, the study demonstrates that serum cortisol is a simple, cost-effective biomarker for risk stratification in AIS. Early identification of patients at higher risk of severe disability or mortality could allow clinicians to optimize monitoring, supportive care, and timely interventions, ultimately improving patient outcomes.

## Conclusions

This study demonstrates that elevated serum cortisol levels are closely linked to greater stroke severity, unfavorable functional outcomes, and higher mortality among patients with acute ischemic stroke. These findings underscore the importance of the HPA axis in stroke pathophysiology and recovery.

However, the results are preliminary and correlational, and they do not establish a direct causal relationship between cortisol elevation and adverse outcomes. Routine clinical application of cortisol measurement as a prognostic tool should be approached with caution until validated by larger, multicenter, and longitudinal studies that adjust for potential confounders. Nevertheless, serum cortisol remains a promising, low-cost biomarker for early risk stratification and could ultimately aid in identifying high-risk patients who may benefit from closer monitoring and targeted interventions.

## References

[1] Ramesh S, Siril N, Rao K C (2023). A study on prognostic value of serum cortisol in stroke and its correlation with NIHSS score. Int J Sci Res.

[2] Zi WJ, Shuai J (2013). Cortisol as a prognostic marker of short-term outcome in chinese patients with acute ischemic stroke. PLoS One.

[3] Barugh AJ, Gray P, Shenkin SD, MacLullich AM, Mead GE (2014). Cortisol levels and the severity and outcomes of acute stroke: a systematic review. J Neurol.

[4] Schoorlemmer RM, Peeters GM, van Schoor NM, Lips P (2009). Relationships between cortisol level, mortality and chronic diseases in older persons. Clin Endocrinol (Oxf).

[5] Mitchell AJ (1997). Clinical implications of poststroke hypothalamo-pituitary adrenal axis dysfunction: A critical literature review. J Stroke Cerebrovasc Dis.

[6] Brott T, Adams HP Jr, Olinger CP (1989). Measurements of acute cerebral infarction: a clinical examination scale. Stroke.

[7] van Swieten JC, Koudstaal PJ, Visser MC, Schouten HJ, van Gijn J (1988). Interobserver agreement for the assessment of handicap in stroke patients. Stroke.

[8] Chobanian AV, Bakris GL, Black HR (2003). Seventh report of the Joint National Committee on Prevention, Detection, Evaluation, and Treatment of High Blood Pressure. Hypertension.

[9] Owolabi MO, Thrift AG, Martins S (2021). The state of stroke services across the globe: Report of World Stroke Organization-World Health Organization surveys. Int J Stroke.

[10] Otte C, Hart S, Neylan TC, Marmar CR, Yaffe K, Mohr DC (2005). A meta-analysis of cortisol response to challenge in human aging: importance of gender. Psychoneuroendocrinology.

[11] Ilanchetchenni K, Chandru J, Kumar SS, Nishanthi G (2024). Serum cortisol levels in assessing severity of acute stroke - a cross-sectional study in Chengalpet Medical College & Hospital. J Popul Ther Clin Pharmacol.

[12] Iranmanesh F, Sedighi B, Ziaadini B (2017). Prognostic value of cortisol in patients with acute ischemic stroke. Zahedan J Res Med Sci.

[13] Agarwal A, Iqbaal H (2020). Association between serum cortisol with severity & prognosis among patients presenting with acute ischaemic stroke. Int J Med Biomed Stud.

[14] Jnanendrappa KH (2019). A study on correlation between serum cortisol and stroke severity with serum cortisol. Academia Journal of Medicine.

[15] Christensen H, Boysen G, Johannesen HH (2004). Serum-cortisol reflects severity and mortality in acute stroke. J Neurol Sci.

[16] Marklund N, Peltonen M, Nilsson TK, Olsson T (2004). Low and high circulating cortisol levels predict mortality and cognitive dysfunction early after stroke. J Intern Med.

[17] Fatima S, Khan R (2020). Prognostic significance of serum cortisol and serum albumin in patients of ischemic stroke. Int J Adv Med.

[18] Saini G, Kaur K, Bhatia L, Kaur R, Singh J, Singh G (2023). Single serum cortisol value as a prognostic marker in acute ischemic stroke. Cureus.

[19] Feigin VL, Brainin M, Norrving B (2022). World Stroke Organization (WSO): Global stroke fact sheet 2022. Int J Stroke.

